# The National Dental Association

**Published:** 1880-07

**Authors:** F. A. Levy

**Affiliations:** General Secretary.


### ARTICLE V.

### *National Dental Association.*

The Executive Committee of the National (late Southern) Dental Association; of the American Dental Association; and of the New York State Society, would respectfully announce to the Dentists of the United States that they hav made ample provision for the reception and entertainment of all Dentists, whether alone or with their families, who will visit New York at the time of the assembling of the Convention from August 9th to 14th. The following hotels are recommended as being favorably suited for the convenience of the Dentists: Sturtevant House, Broadway, near 28th Street; Coleman House, Broadway, near 29th Street; Continental Hotel, cor. Broadway and 20th Street; Rossmore Hotel, cor. Broadway and 42d Street; Grand Central Hotel, Broadway and 3d Street. The above Hotels will accommodate guests with rooms at the rate of $1 per night for each person, and $2.50 for room and board for each person. Parlors have been offered at Sturtevant House, Coleman House and at the Continental for the use of the Committees. On and after the morning of August 9th, a Committee will be in attendance at Sturtevant House for the purpose of giving visitors information on all points connected with the convention.

Excursion Fares.—The railroad excursion arrangements by the lines from the East, West and Northwest are so ample and the rates so low, that it is not necessary to make special arrangements.

From Southern points the excursion rates to New York and return will be as follows: Norfolk, Va., by steamer, $14; Charleston, S. C., by steamer, $25; Augusta, Ga., via. Charleston steamer, $30; Columbia S. C.,via. Charleston steamer, $30; Atlanta, Ga., via. Charleston Steamer, $40.35, Macon, Ga., at the rate of 6c. per mile, one way, to Augusta, thence by Cahrlestown. Other railroad rates not yet arranged for will be reported through the Presidents of the several State societies.

There will be several excursions given by the Dentists of New York and vicinity, in which all visiting Dentists are invited te participate with their families, which will, no donbt add to the pleasure of the meeting.

(Signed,) F. A. Levy,
*General Secretary.*

